# Current biologics in treatment of pemphigus foliaceus: a systematic review

**DOI:** 10.3389/fimmu.2023.1267668

**Published:** 2023-10-12

**Authors:** Caden A. Carver, Mikole Kalesinskas, A. Razzaque Ahmed

**Affiliations:** ^1^ Midwestern University, Arizona College of Osteopathic Medicine, Glendale, AZ, United States; ^2^ Department of Dermatology, Center for Blistering Disease, Tufts University School of Medicine, Boston, MA, United States; ^3^ Department of Dermatology, Tufts University School of Medicine, Boston, MA, United States

**Keywords:** pemphigus foliaceus, pemphigus vulgaris, rituximab, intravenous immunoglobulin, biologics, systemic corticosteroids, immunosuppressive agents

## Abstract

**Background:**

Pemphigus foliaceus (PF) differs from pemphigus vulgaris (PV) in that it affects only the skin and mucous membranes are not involved. Pemphigus is commonly treated with systemic corticosteroids and immunosuppressive agents (ISAs). More recently, biologics have been used. The current literature on biologic therapy often combines treatment of PF with PV, hence it is often difficult for clinicians to isolate the treatment of PF from PV. The purpose of this review was to provide information regarding the use of current biological therapy, specifically in PF.

**Materials and methods:**

A search of PubMed, Embase, and other databases was conducted using keywords pemphigus foliaceus (PF), rituximab (RTX), intravenous immunoglobulin (IVIg), and biologics. Forty-one studies were included in this review, which produced 105 patients with PF, treated with RTX, IVIg, or a combination of both. Eighty-five patients were treated with RTX, eight patients with IVIg, and 12 received both RTX and IVIg.

**Results:**

Most patients in this review had PF that was nonresponsive to conventional immunosuppressive therapies (CIST), and had significant side effects from their use. RTX treatment resulted in complete remission (CR) in 63.2%, a relapse rate of 39.5%, an infection rate of 19.7%, and a mortality rate of 3.9%. Relapse was greater in the lymphoma (LP) protocol than the rheumatoid arthritis (RA) protocol (p<0.0001). IVIg led to CR in 62.5% of patients, with no relapses or infections. Patients receiving both biologics experienced better outcomes when RTX was first administered, then followed by IVIg. Follow-up durations for patients receiving RTX, IVIg, and both were 22.1, 24.8, and 35.7 months, respectively.

**Discussion:**

In pemphigus foliaceus patients nonresponsive to conventional immunosuppressive therapy or in those with significant side effects from CIST, RTX and IVIg appear to be useful agents. Profile of clinical response, as well as relapse, infection, and mortality rates in PF patients treated with RTX were similar to those observed in PV patients. The data suggests that protocols specific for PF may produce better outcomes, less adverse effects, and improved quality of life.

## 1 Introduction

Pemphigus is an autoimmune mucocutaneous blistering disease that has two major subsets. The more common Pemphigus vulgaris (PV) involves mucous membranes, in addition to the skin, and is characterized by intraepidermal vesicles, deposition of IgG and C3 on the cell surface of the entire epidermis, and autoantibodies to both desmoglein 1 and 3 (1, 2). Pemphigus foliaceus (PF) is limited to the skin. It is characterized by subcorneal vesicles on histology and IgG and C3 deposition in the superficial epidermis on direct immunofluorescence (DIF) (1, 2). Sera of PF patients has antibodies to desmoglein 1 only (1, 2). It needs to be emphasized that the incidence of PF is considerably lower than that of PV, and that PF is clinically distinct from PV.

The vast majority of studies on pemphigus have combined PF patients with PV patients. Since there are considerable differences between the two, the authors in this review have presented data on the clinical outcomes solely in PF patients. The important reason for this was, when a treating physician decides to use a biologic agent to treat PF, this systemic review may facilitate the decision making process.

Since the advent of systemic corticosteroids, pemphigus patients have been treated with these potent anti-inflammatory agents (1, 2). The significant side effects of systemic corticosteroids led to the use of immunosuppressive agents (ISAs), with the goal being to reduce autoantibody production and have a possible steroid-sparing effect. However, the combination of systemic corticosteroids and ISAs as conventional immunosuppressive therapy (CIST) contributed to profound immunosuppression, leading to opportunistic infections and death in some cases (2). Hence, newer therapeutics were needed.

In an attempt to reduce the adverse events associated with CIST, biologic agents have been introduced in treating pemphigus (1, 2). Biologic agents are pharmacological agents derived from human plasma or produced by cells *in vivo* in a laboratory using a spectrum of technologies of molecular biology. In this review, only use of two such agents are described. Intravenous immunoglobulin (IVIg) is derived from human plasma. Rituximab (RTX) is a half human, half mouse monoclonal antibody, which targets CD20^+^ B-cells. Initially, IVIg was used. More recently, the use of RTX, a B-cell depleting agent, has been advocated by several authors as first line therapy for pemphigus (2). In these studies, the emphasis has been on PV, and the readership often does not get the opportunity to isolate specific responses in PF patients.

Therefore, in this review, the authors focus solely on presenting data on patients with PF, treated with RTX and IVIg. The main purpose of this review is to provide specific information on the clinical outcomes and adverse events associated with the use of only these two biologic agents in PF. As present in current literature, this could result in evidence-based clinical decisions that may possibly benefit physicians and patients.

## 2 Materials and methods

**Figure 1:**
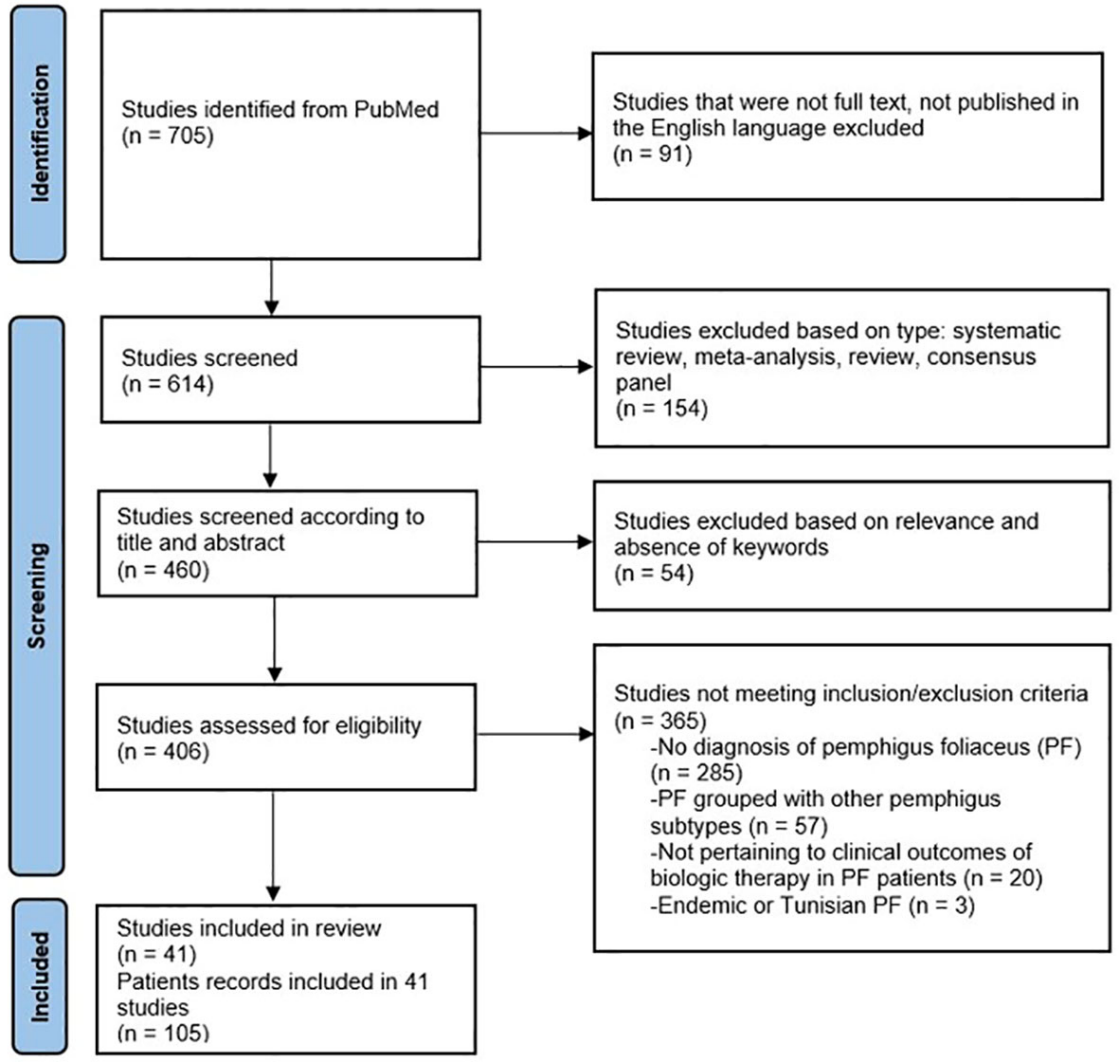
PRISMA flow diagram for systematic review of current biologics in treatment of pemphigus foliaceus (PF).

Collection of the data for this analysis is presented in the PRISMA flow diagram (**Figure 1**). A review of PubMed, Embase and other databases was conducted from January 1990 to July 2023, using keywords pemphigus foliaceus, rituximab, intravenous immunoglobulin (IVIg) and other biologic agents. Forty-one studies (3–43) were included in this review, totaling to 105 patients with PF, treated with a biologic agent/(s). Eighty-five adult patients received RTX, and eight received IVIg. Twelve patients received a combination of RTX and IVIg.

Inclusion criteria were (1) patients with PF diagnosed by clinical presentation, histology, and direct immunofluorescence, (2) patients treated with biologic agents, and (3) studies published in the English language. Exclusion criteria were (1) studies failing to differentiate pemphigus foliaceus from pemphigus vulgaris, and (2) studies where a joint diagnosis of PF and PV was made, (3) pediatric patients, (4) endemic PF, and (5) Tunisian PF patients.

Data extracted from each study, when present, included patient demographics, duration of disease prior to therapy, previous and concomitant therapies, treatment protocol, clinical outcomes, length of follow-up, relapse, adverse events, CD 20^+^ B-cell studies, and anti-Dsg1 antibody levels. Statistical analyses were performed using MedCalc for Windows, version 22.009 (MedCalc Software, Ostend, Belgium).

## 3 Results

### 3.1 Patient characteristics

#### 3.1.1 Rituximab group

Eighty-five patients were treated with RTX. Of the 85 patients, 50% were female and 50% were male. The mean age at initiation of RTX was 51.4 years (range 21-86). RTX was initiated at a mean of 55.4 months (range 0-540) after diagnosis of PF. Comorbidities were reported in eight (9.4%) patients. Of these, two had diabetes mellitus not attributed to previous PF treatment (3, 4), two were obese (3, 4), two had hypertension (3, 4), and two had Hepatitis C (5, 6). Other comorbidities included autoimmune hepatitis (7), hypothyroiditis (8), previously resected C-kit positive gastrointestinal stromal tumor and urothelial cell bladder cancer (8), tobacco use (9), gastroesophageal reflux disease (GERD) (9), dyslipidemia (10), history of pulmonary embolism (10), and anemia (11). Most patients had severe or recalcitrant disease or contraindications to conventional immunosuppressive therapy.

#### 3.1.2 IVIg group

Eight patients were treated with IVIg. Of the 8 patients, 66.7% were female and 33.3% were male. The mean age at IVIg initiation was 50 years (range 31-66). Comorbidities were reported in one patient, who had Sjögren’s syndrome and asthma (12). In all patients the indication for IVIg was disease nonresponsive to conventional immunosuppressive therapy and side effects from its use. The mean duration of PF prior to IVIg was 83 months (range 82-84).

#### 3.1.3 RTX and IVIg group

Twelve patients received both RTX and IVIg. Of the 12 patients, 41.7% were female and 58.3% were male. The mean age of patients receiving RTX and IVIg was 58.8 years (range 38-77). The mean duration of PF at initiation of therapy was 52.5 months (range 12-132). No comorbidities were reported by the authors in the RTX and IVIg group.

The authors presume that the dermatologists that initially used IVIg, did so because it may be the only biologic agent available at that time. It is entirely possible that IVIg did not produce the desired clinical outcome. Now, as RTX was available, it was used. The authors who used RTX and IVIg simultaneously may have done so because they presumed that the combination would be clinically effective. The treating dermatologists who used RTX initially and subsequently used IVIg may have done so because the patient may have had a relapse after RTX use.

It needs to be highlighted that none of the authors of these publications provided any reasoning for the sequence in which they used these biologic agents.

### 3.2 Previous therapies

#### 3.2.1 Rituximab group

Previous therapies are reported in 81 patients (95.3%) (**Table 1**). Seventy-four (91.3%) received corticosteroids. Of those, 66 (81.5%) received oral corticosteroids and 19 (23.5%) received intravenous corticosteroids. Sixty-two (76.5%) received both corticosteroids and immunosuppressive agents (ISAs). Of sixty-two (76.5%) patients treated with ISAs, 50 (61.7%) received azathioprine, 21 (25.9%) received mycophenolate mofetil, eight (9.9%) received cyclophosphamide, three (3.7%) received methotrexate. Anti-inflammatory agents were also used. One (1.2%) received gold (13), one (1.2%) received hydroxychloroquine (7), and dapsone was used in thirteen patients (16%).

**Table 1:** Previous therapies in patients receiving rituximab or intravenous immunoglobulin.

Previous therapies	Rituximab N=81 (%)*	IVIg N=8 (%)
Corticosteroids	74 (91.4%)	8 (100%)
Azathioprine	50 (61.7%)	4 (50%)
Mycophenolate mofetil	21 (25.9%)	1 (12.5%)
Dapsone	13 (16%)	2 (25%)
Cyclophosphamide	8 (9.9%)	1 (12.5%)
Methotrexate	3 (3.7%)	2 (25%)
Gold	1 (1.2%)	None
Hydroxychloroquine	1 (1.2%)	1 (12.5%)

*In 4 patients treated with rituximab, the authors mentioned prior use of conventional immunosuppressive therapy (CIST), but did not provide details of which agent/(s) were used. Hence, the number of patients is n=81.

IVIg, intravenous immunoglobulin.

Adverse effects of previous therapy were reported for 16 (19.8%) patients. Nine (11%) patients developed Cushing syndrome or steroid-induced diabetes. Osteoporosis, cytopenia, and hepatotoxicity were reported in four (4.9%) patients each. Additional side effects included gastrointestinal hemorrhage (10), eczema herpeticum (4), ophthalmological disorders (14), myalgia (15), and trembling (15).

#### 3.2.2 IVIg group

Previous therapies are presented in **Table 1**. All patients received oral corticosteroids and one (12.5%) received intravenous corticosteroids. Four (50%) were treated with ISAs, of which all received azathioprine. Two (25%) patients received methotrexate, two (25%) received dapsone (12, 16), one (12.5%) received mycophenolate mofetil (16), one (12.5%) received cyclophosphamide (12), and one (12.5%) received hydroxychloroquine (12). Tóth et al. reported one patient treated unsuccessfully with 10 courses of plasma exchange (12).

Adverse effects were reported for two (25%) patients, one (12.5%) who experienced gastrointestinal hemorrhage while receiving corticosteroid therapy (16), and one (12.5%) who developed anemia secondary to dapsone (17).

#### 3.2.3 RTX and IVIg group

Prior to receiving both RTX and IVIg, all 12 patients were treated with systemic corticosteroids or ISAs, or a combination of both. ISAs used were azathioprine, mycophenolate mofetil, methotrexate, cyclophosphamide, and cyclosporine. Additional agents used were dapsone, hydroxychloroquine, gold, and plasmapheresis.

### 3.3 Treatment regimen

#### 3.3.1 Rituximab group

Eighty-five PF patients received RTX therapy. Thirty-nine (45.9%) patients were treated with the rheumatoid arthritis (RA) protocol (two 1000 mg infusions two weeks apart) and 42 (49.4%) were treated with the lymphoma protocol (LP) (four 375 mg/m2 infusions one week apart). Two (2.4%) patients received RTX via an unspecified protocol (18) and two (2.4%) patients received RTX as maintenance therapy with a single 1000 mg infusion every six months (19). RTX was used as first-line therapy in four (4.9%) patients.

Concomitant therapy was reported in all 85 patients (100%) (**Table 2**). Systemic corticosteroids were given in 48 (56.5%) patients, azathioprine in 22 (25.9%) patients, and mycophenolate mofetil in eight (9.4%) patients. Three (3.5%) patients received dapsone (15, 20, 21) with RTX. Additional therapies given with RTX were protein A immunoadsorption (PAIA) (6, 21), cyclosporine (22), and imatinib (8).

**Table 2:** Concomitant therapies with rituximab or intravenous immunoglobulin.

Concomitant therapies	Rituximab N=85 (%)	IVIg N=8 (%)
Corticosteroids	48 (56.5%)	8 (100%)
Azathioprine	22 (25.9%)	None
Mycophenolate mofetil	8 (9.4%)	None
Dapsone	3 (3.5%)	4 (50%)
Protein A Immunoadsorption (PAIA)	2 (2.4%)	None
Cyclosporine	1 (1.2%)	None
Imatinib	1 (1.2%)	None

IVIg, intravenous immunoglobulin.

#### 3.3.2 IVIg group

Eight adult PF patients received IVIg therapy. Protocols varied, but IVIg was most-commonly given at 0.4 g/kg/day for five consecutive days (17, 23). The number of IVIg cycles was not frequently reported.

All eight (100%) patients received concomitant therapy with corticosteroids. Four (50%) received dapsone, and one (12.5%) received pulsed dexamethasone (12). (**Table 2**).

#### 3.3.3 RTX and IVIg group

In those receiving both agents, two (16.7%) patients were given RTX prior to IVIg. One of these patients was treated with the LP protocol, and one was treated with an unspecified protocol. Two (16.7%) were given RTX and IVIg concomitantly. Both received the rheumatoid arthritis (RA) protocol. Eight (66.7%) patients were given IVIg prior to RTX. Five were treated with the LP protocol, one was treated with the RA protocol, and two received unspecified RTX protocols. All patients received additional therapy with conventional ISAs.

### 3.4 Clinical outcomes

#### 3.4.1 Rituximab group

Clinical outcomes were analyzed for the 81 (95.3%) patients treated with RTX, using the LP or RA protocol. The course of these patients is presented in **Table 3**. Of these 81 patients, clinical outcomes were not reported for five patients. Thus, clinical outcomes were analyzed for 76 patients, of whom 74 (97.4%) patients experienced clinical improvement. Treatment failure was experienced by one (2.6%) patient receiving the RA protocol (10) and one (2.6%) patient receiving the LP protocol (13). Rates of complete remission (CR), CR off-therapy, CR on-therapy, nonresponse, and follow-up were statistically similar between the two (**Table 3**). Relapse occurred significantly more in the LP protocol than the RA protocol (68.4% vs 10.5%, p<0.0001). Forty-eight (63.2%) of all patients treated with RTX achieved CR. Remission status on or off-therapy was not reported for two patients treated with RTX via the RA protocol, however, both reached CR. Thus, these patients were included in the total number of patients achieving CR. Twenty-two (28.9%) achieved CR off-therapy and 24 (31.6%) achieved CR on-therapy (**Table 3**). Twenty-two (28.9%) reached partial remission (PR).

**Table 3:** Clinical outcomes in 76 patients treated with rituximab, using the rheumatoid arthritis (RA) protocol or lymphoma protocol (LP).

Protocol (# patients)*	CR % (n)**	CR off % (n)	CR on % (n)	PR % (n)	Nonresponse/failure (n)	Relapse % (n)	Follow-up, months (range)
**RA (38)**	60.5% (23)	23.7% (9)	31.6% (12)	28.9% (11)	2.6% (1)	10.5% (4)	21.9 (4-84)
**LP (38)**	65.8% (25)	34.2% (13)	31.6% (12)	28.9% (11)	2.6% (1)	68.4% (26)	22.2 (4-75)
**P-value**	NS	NS	NS	NS	NS	p<0.0001	NS

*Clinical outcomes were not reported in five out of 81 patients treated with RTX via the RA or LP protocols. Hence, data is provided only on 76 patients.

**Number of patients reaching complete remission (CR) included those reaching CR on or off-therapy, and those where on or off-therapy was not specified in reaching CR.

CR, complete remission; CR off, clinical remission off-therapy; CR on, clinical remission on-therapy; PR, partial remission; RA protocol, rheumatoid arthritis protocol; LP, lymphoma protocol; NS, not statistically significant.

#### 3.4.2 IVIg group

All patients reported clinical improvement. Five (62.5%) total patients reached CR, including four (50%) who reached CR on-therapy. One patient reached CR, but it was not specified if the patient was in remission on or off-therapy. Two (25%) patients reached PR. One patient had clinical improvement without meeting the criteria for PR or CR and is not included in **Table 4**. Clinical outcomes are reported in **Table 4**.

**Table 4:** Clinical outcomes after rituximab, via the LP or RA protocols, or intravenous immunoglobulin.

Protocol (# patients)	CR % (n)	CR off % (n)	CR on % (n)	PR % (n)	Nonresponse/failure % (n)	Relapse % (n)	Infection % (n)	Mean follow-up, months (range)
**RTX (n=76) ^§^**	63.2% (48)*	28.9% (22)	31.6% (24)	28.9% (22)	2.6% (2)	39.5% (30)	19.7% (15)	22.1 (2-84)
IVIg (n=8)**	62.5% (5)	None	50% (4)	25% (2)	None	None	None	24.8 (6-51)
**p-value**	NS	p<0.0001	NS	NS	p<0.0001	p<0.0001	p<0.0001	NS

**^§^** Clinical outcomes were not reported in five out of 81 patients treated with RTX via the RA or LP protocols.

*The total number of patients reaching CR (48) included patients reaching CR-off and on therapy. In two reports, the authors of those reports did not specify if the patient was on or off therapy, and were thus included in the total value reaching CR.

**One patient only had clinical improvement, without meeting criteria for either CR or PR, and was thus not presented here.

CR, complete remission; CR off, clinical remission off-therapy; CR on, clinical remission on-therapy; PR, partial remission; RA protocol, rheumatoid arthritis protocol; LP, lymphoma protocol; NS, not statistically significant.

#### 3.4.3 RTX and IVIg group

Six (54.5%) patients had CR, and five (45.5%) had PR. Clinical outcome was not reported for one patient. Outcomes are reported in **Table 5**.

**Table 5:** Clinical outcomes after rituximab and intravenous immunoglobulin therapy.

Regimen	CR, n (%)	PR, n (%)	Relapse, n (%)	Infection, n (%)
CIST→RTX→IVIg* **N=2**	1 (50%)	1 (50%)	None	None
**CIST→RTX+IVIg** **N=2**	1 (50%)	1 (50%)	None	1 (50%)
**CIST→IVIg→RTX** **N=8**	4 (57.1%) *	3 (42.9%) *	4 (50%)	2 (25%)

*One patient did not report the final outcome as CR or PR, but only clinical improvement.

Hence, the number of patients in **Table 3** is 11.

CIST, conventional immunosuppressive therapy; RTX, rituximab; IVIg, intravenous immunoglobulin; CR, complete response; PR, partial response.

### 3.5 Follow-up duration

#### 3.5.1 Rituximab group

Patients were followed for a mean of 22.1 months (range 2-84) (**Table 4**).

#### 3.5.2 IVIg group

Patients were followed for a mean of 24.8 months (range 6-51) (**Table 4**).

#### 3.5.3 RTX and IVIg group

Patients were followed for a mean of 35.7 months (range 5-110).

### 3.6 Relapse

#### 3.6.1 Rituximab group

Thirty relapses occurred in 22 patients, with a relapse rate of 39.5%. In 18 relapses, time course was reported. Relapse occurred at a mean of 20.7 months (range 1.4-84) after last RTX infusion. In two patients, B-cell repopulation occurred at 6.5 and 10 months, followed by relapses at 9 and 13 months, respectively (24). Two patients relapsed when CD20^+^ cells were less than 10% (22). In non-relapsing patients, time course for B-cell depletion was reported in four patients. Cianchini et al. reported two patients in whom B-cell depletion occurred within the first RTX infusion and remained undetectable for at least six months (25). Marzano et al. reported two patients in whom CD20^+^ depletion occurred at three months and lasted for 15-18 months (26). Anti-Dsg1 levels were reported during relapse in two patients (8, 27). Antibodies in both were negative following therapy with RTX. In one, anti-Dsg1 level was 75 U/mL when relapse occurred after 84 months (27). In the other, anti-Dsg1 level was 27.25 U/mL when a relapse occurred after 42 months (8).

Treatment of relapse was reported for 15 of 22 patients. Nine (60%) received additional RTX, seven (46.7%) received corticosteroids, two (13.3%) received dapsone, and one (6.7%) received azathioprine. Following relapse, 14 patients (63.6%) again achieved disease control. Eight (36.4%) patients had CR and three (13.6%) had PR.

#### 3.6.2 IVIg group

No relapses were observed in patients receiving IVIg therapy.

#### 3.6.3 RTX and IVIg group

Four (33.3%) of 12 patients receiving both IVIg and RTX therapy relapsed (**Table 5**). All relapses occurred in patients who first received IVIg, followed by RTX. Patients who relapsed were all given additional RTX. In one patient only partial response was achieved. In two, clinical remission off- therapy was reported. In patients who got IVIg after RTX and those who got both agents simultaneously, relapses were not reported during the follow-up period. Clinical response was not reported in one patient. B-cell studies were not reported in this group.

### 3.7 Adverse effects

#### 3.7.1 Rituximab group

Side effects were reported in 18 (23.7%) patients. Of these side effects, 15 episodes of infection occurred in 13 patients (**Table 6**), resulting in an infection rate of 19.7% (**Table 4**). Infection occurred at a mean of 3.3 months (range 0-10) after treatment with RTX. All patients received corticosteroids during treatment. At the time of infection, nine (69.2%) patients were receiving either systemic corticosteroids or ISAs for maintenance therapy. Six (46.2%) were on systemic corticosteroids, four (30.8%) were on azathioprine, and one (7.7%) was on mycophenolate mofetil. Three (23.1%) patients were on both steroids and ISAs and two (15.4%) were on more than one ISAs when infection was reported (**Table 6**).

**Table 6:** Infections in 76 patients treated with rituximab via the LP or RA protocols.

Infection	Number of infections N=76 (%)^§^	Duration since last RTX	Concomitant therapies
Disseminated nocardiosis with widespread abscesses leading to death	1 (1.3%)	1 wk	pred
*Pneumocystis jirovecii* pneumonia	1 (1.3%)	6 m	psl, aza
Joint infection with septic shock	1 (1.3%)	2 wk	psl, aza
Osteomyelitis	1 (1.3%)	4 m	psl
Erysipelas	2 (2.6%)	3 wk, 10 wk	dap, aza
*Staph*y*lococcus aureus* skin infection/impetigo	3 (3.9%)	NR	pred, dap
Herpes Simplex	1 (1.3%)	NR	NR
Upper respiratory tract infection	1 (1.3%)	NR	NR
Herpes Zoster	1 (1.3%)	0 wk	mmf
COVID-19 pneumonia	1 (1.3%)	10 m*, 2 m**	pred
Viral conjunctivitis	1 (1.3%)	NR	NR
Dental abscess	1 (1.3%)	4 m	psl, aza

**^§^** Clinical outcomes were not reported in five out of 81 patients treated with RTX via the RA or LP protocols.

*After initial rituximab.

**After repeat rituximab.

Psl, prednisolone; pred, prednisone; aza, azathioprine; mmf, mycophenolate mofetil; dap, dapsone; NR, not reported. *after initial rituximab, **after repeat rituximab.

Mortality occurred in three (3.9%) patients treated with RTX. One patient had disseminated nocardiosis (10), and one had osteomyelitis and gastrointestinal hemorrhage (20). In one patient the specific cause of death was not reported (13). This patient had relapsed and received additional RTX and systemic corticosteroids during their course of therapy (13). It is important to note that gastrointestinal hemorrhage could be related to concomitant therapy, and not RTX therapy. Furthermore, osteomyelitis can occur as a consequence of RTX and concomitant therapy.

#### 3.7.2 IVIg group

A mild infusion reaction occurred in one patient. Adverse effects and mortality were not reported in patients treated with IVIg. Maintenance therapy following IVIg was reported for six (75%) patients. Four (50%) were on systemic corticosteroids and three (37.5%) were on dapsone, with one (12.5%) receiving both dapsone and systemic steroids (17).

#### 3.7.3 RTX and IVIg group

Infection was reported in three (25%) patients treated with RTX and IVIg (**Table 5**). Of these, one (8.3%) received RTX and IVIg simultaneously. This patient had both erysipelas and multiple HSV infections (15). One patient had a labial abscess and a VZV infection (15). One had tuberculous meningitis after RTX (13). These patients received both systemic corticosteroids and ISAs during treatment with biologics (13, 15).

## 4 Discussion

Clinical studies and reviews on the use of biologics in pemphigus, mix patients with PF and PV, though the two are clinically, histologically, and immunopathologically distinct. In order to better understand the role of these treatments in PF, the authors segregated patients with PF from those with PV and presented their clinical outcomes.

To analyze the clinical response to biologic agents in PF, patients were divided into three groups. Eighty-five patients were treated with rituximab (RTX). Eight patients were treated with intravenous immunoglobulin (IVIg). Twelve patients were treated with RTX and IVIg. Indications for the use of biologic therapy were disease that was nonresponsive to conventional immunosuppressive therapy (CIST) or previous adverse effects from CIST. In this review, only use of two such biologic agents were analyzed. In the literature, only two cases of the use of TNFα inhibitors in PF are reported (44, 45). Due to this limited number of patients, the authors did not include it as a major biologic agent to treat PF in this review. It is important to highlight that, like PF, these agents have been used in a very limited number of patients with PV also. Future studies of large cohorts of PF patients treated with other biologics, such as etanercept, are necessary to establish their efficacy in treatment of PF.

RTX resulted in complete remission in 48 patients (63.2%), although 30 patients (39.5%) relapsed, and the infection rate was 19.7%. The mortality rate was 3.9%. The clinical outcomes of the rheumatoid arthritis (RA) and lymphoma (LP) protocols were similar, although less relapses were reported when the RA protocol was used. The mean duration of follow-up for this group was 22.1 months (range 2-84).

Four patients received RTX as first line therapy (24, 28). Three of these patients relapsed and required additional RTX and systemic corticosteroids (24). Only one patient had sustained clinical remission (28). Treatment with IVIg resulted in complete remission in 5 patients (62.5%), which was sustained and without adverse effects. No infection or mortality was reported in this group in a follow-up of 24.8 months. At the time of reporting, patients were still on IVIg therapy. Hence, a true relapse rate could not be determined from the available data.

Clinical outcomes of a combined protocol with RTX and IVIg varied depending on the order in which each biologic was used. In this group, relapse occurred only when RTX was used as the second biologic, after initial therapy with IVIg. Two out of three infections occurred when RTX was given as the second biologic, after IVIg. In contrast, when RTX was used as the first biologic agent, followed by the addition of IVIg, there was no relapse or infection. Duration of follow-up was 35.7 months (range 5-110).

Patients in all three groups were frequently treated with systemic corticosteroids and ISAs as concomitant therapy and post-biologics as maintenance therapy. Consequently, two issues emerged. The simultaneous use of CIST increased immunosuppression, which may have resulted in systemic infections and mortality. The use of CIST also made it difficult to isolate the direct effect of each biologic agent.

Chen et al. reported that 90% of PV patients treated with RTX and systemic corticosteroids reached clinical remission (CR) off-therapy (46). A 2015 review of 433 PV patients reported CR off-therapy in 57.6% with the LP protocol and 47.3% with the RA protocol (47). PV patients had relapse rates of 65% after the RA protocol and 40.7% after the LP protocol (47). Infections occurred in 5% (48), 16.9% (49), 25% (50), and 63% (51) of patients following RTX. Mortality rates as high as 5.2% were reported in pemphigus patients treated with RTX (48). In most, but not all, the cause of death was infection leading to septicemia.

Previous reports indicate that the relapse rate after RTX is dependent on the duration of follow-up (52). This was demonstrated in a French study of PV patients, wherein a relapse rate of 77.3% was reported in a follow-up of 6.6 years after RTX (52).

Adverse event profiles varied in other autoimmune diseases treated with RTX. Some examples are as follows. Serious infection occurred in 2.3% of rheumatoid arthritis patients (53), 15.4% of autoimmune vasculitis patients (54), and 23.7% of relapsing-remitting multiple sclerosis patients (55). In monoclonal hematologic disease, serious infection was reported in 58%, with a mortality rate of up to 35.4% following RTX therapy (56). Serious infections and death are of grave concern to physicians, patients, and their families.

IVIg has demonstrated clinical effectiveness with minimal adverse events in previous studies on PV (57). A 2001 study reported 15 patients with recalcitrant PV, treated with IVIg as monotherapy, resulting in sustained clinical remission without relapse or adverse effects over a follow-up of 20.4 months after IVIg (57). In a Consensus Developing Conference, which included 38 experts on blistering disease from the United States, Canada, and Europe, a succinct protocol was described for the use of IVIg in autoimmune blistering disease. According to these consensus guidelines, CIST may be discontinued, followed by IVIg as monotherapy for successfully achieving complete remission (58).

Previous studies have reported combination therapy with RTX and IVIg to successfully treat PV patients. A 2006 study described a protocol in which the combination of RTX and IVIg produced clinical remission in a cohort of patients with recalcitrant PV (59). Initially, multiple doses of RTX were used to deplete autoreactive clones of autoantibody producing B-cells. IVIg was then added for immunoprophylaxis and subsequently to promote immune balance and reconstitution (59). Patients had no infections, hospitalizations, and no mortality (59). In a subsequent publication by the same authors, these patients were drug-free, disease-free, and had sustained clinical remission 15 years later (60). A study of 10 patients with PV, in whom the use of systemic corticosteroids and ISAs was strongly contraindicated, reported the combination protocol of RTX and IVIg, used as first-line therapy (61). These patients remained in clinical remission with no disease and no drugs for 7.2 years after the last RTX infusion (61).

IVIg demonstrated beneficial outcomes in the treatment of PF in this study, and in previous studies on PV. Nonetheless, IVIg has been used considerably less than RTX. One of the reasons for the less frequent use of IVIg could be the perceived high cost associated with it. The high price is entirely due to the cost of producing the drug. However, a 2006 study described that the total cost of IVIg was considerably lower than the cost of CIST in treating autoimmune bullous diseases (62). The authors created the paradigm that the total cost of a drug includes the cost of the drug itself, in addition to the cost of treating side effects it produces (62). If that paradigm is applied to comparing the cost of IVIg to RTX, the true cost of RTX would likely be higher, but impossible to fully assess. While determining the cost of adverse effects such as infection and associated hospitalization is possible, the cost of death can never be truly quantified. In a 2017 study by a French group in Lancet, RTX was reported as being less expensive than IVIg and was promoted as first-line therapy (63). The follow-up period of this study was very limited and coincided with recent RTX infusions (63). No deaths were reported by the authors. The study was funded by the manufacturer and therefore no cost was incurred by the patients (63). Furthermore, it was not clarified that healthcare in most of Europe is state funded, and patients frequently do not bear the full burden of treatment costs. In many European countries, permissions and authorizations for the use of biologic agents are made by local health authorities. Healthcare in the United States is funded by private health insurance companies and the State pays only for the elderly or disabled. Funding for health is variable worldwide. Hence, cost comparisons are likely to be effective only locally.

There were several limitations to this study. A major limitation was the lack of a control group. In a rare, potentially fatal disease, this may not be possible, and may be considered unethical. Since the prevalence of PF is low, patient cohorts were limited in size. Eighty-five patients were treated with RTX and only eight patients were treated with IVIg, therefore, a critical comparison of many parameters becomes difficult and can be biased based on size. Another major concern was the lack of detailed clinical information and a limited follow-up period. Additionally, infrequent reporting of CD20^+^ B-cell counts before, during, and after RTX therapy limited the ability of the study to determine B-cell depletion and repopulation. The infrequent reporting of autoantibody titers to desmoglein-1 was also a significant limitation. Both of these aspects prevented the correlation of clinical response, and more importantly, relapse, with repopulation of B-cells and rise in autoantibody titers. In the final analysis, it was somewhat difficult to fully assess the impact of these new therapies on the B-cell biology and autoantibody production, besides the clinical course of disease.

The clinical presentation, histology, and immunopathology of PF is different from PV. Therefore, it would be reasonable to suggest that protocols using biologic agents or other systemic therapies to treat PF should be different from PV. Furthermore, since PF only involves the skin, it would be reasonable to propose that topical therapy be implemented in a manner that decreases the need for high doses of systemic therapy. In addition, most PF lesions occur due to involuntary trauma. Therefore, advising patients to avoid such trauma will lead to better disease control and improved quality of life. There is evidence that extensive UV exposure may also trigger the formation of PF lesions (64, 65). Hence, patients must be advised about photoprotection as an added precaution.

A combination of these conservative management strategies may, to some degree, limit the use of systemic therapies. When PF lesions are severe, widespread, or nonresponsive to conservative management, biologic therapy should be applied systematically. RTX is initially effective at depleting autoreactive B-cells that produce autoantibodies. Excessive immunosuppression may not be in the best interest of the patient. IVIg provides immunoprophylaxis and may be beneficial whenever its use is possible. Since there was such a vast disparity in the number of patients treated with RTX compared to IVIg, it would be unreasonable to provide an opinion on which biologic agent is preferably recommended. IVIg appears safe, has fewer adverse events, no infections and no relapses. However, it is often cost prohibitive. Randomized control trials (RCT) for the use of IVIg in PV or PF are unlikely because most of the IVIg produced is rapidly used. Not infrequently, it is often unavailable. There is no incentive to the manufacturers to do a RCT. In the U.S., its use in PV and PF is covered by Medicare and most private insurance companies. The authors recognize that in some developing countries, it may not be available. When available, it may not be affordable. Rituximab is cheaper but has its own baggage. According to the updated guidelines provided by the European Academy of Dermatology and Venereology (EADV) (66), the first-line treatments for PF include dapsone, corticosteroids, or RTX (RA protocol). However, these guidelines are intended for dermatologists treating PF in Europe. Treatment of patients in most European countries is state sponsored. This may not be the case for the rest of the world. Consequently, in the United States, as well as globally, there is no uniform policy or guidelines of how to treat PF. Hence, the treating dermatologist will need to consider multiple factors in deciding which agent to choose or to use both agents. Recognizing the rarity of PF, multicentered trials that provide statistically significant numbers of patients are needed to develop comprehensive therapeutic approaches and management protocols. Future research should target such a goal.

In conclusion, the data analysis in this review suggests that rates of clinical response, relapse, infection and mortality in PF patients, treated with RTX, are reasonably similar to the majority of PV patients treated with RTX. RTX is an effective agent for PF. In spite of differences between PV and PF, similar results were observed when RTX was used. There could be many reasons. One important reason is that both diseases are characterized by autoantibodies to desmogleins, produced by autoreactive B-cells. RTX kills these cells in both clinical entities with similar efficacy and efficiency. The RA and LP protocols led to similar rates of clinical response, although less relapses occurred with the RA protocol. Infection was common after RTX, suggesting that measures need to be taken to protect patients when this therapy is used. Although the cohort was limited, IVIg resulted in similar clinical efficacy to RTX with no relapses, infections, or hospitalizations. Considering these outcomes, it seems reasonable to recommend a combination of RTX and IVIg when PF is severe, widespread, or recalcitrant and combination therapy is possible or permitted.

This review reinforces that PF is clinically distinct from PV, in that it involves only the skin and not the mucous membranes. The histology and immunopathology is limited to the very upper layers of the epidermis. The predominant autoantibody targets only desmoglein-1. Therefore, it would be reasonable to presume that the autoreactive cells that produce the autoantibody may be different in PF than PV. Likewise, the molecular mechanisms in the pathogenesis and in the microenvironment could be different in the two distinct forms of pemphigus.

As such, it would be in the best interest of both the patients and their physicians for protocols specific to PF, to be designed, developed, and effectively used. The main reason to suggest such an action is that only cutaneous disease may require less aggressive therapy. In PV patients, it is often that cutaneous disease responds more rapidly than mucosal disease. Cutaneous disease lends itself to topical therapy, which, if effective, may warrant less systemic therapy. It is entirely possible that PF patients may encounter less adverse effects. Using new ideas and creating new treatment protocols is progressive clinical medicine, which brings new advances and opens corridors for new thoughts and ideas.

## Data availability statement

The original contributions presented in the study are included in the article/supplementary material. Further inquiries can be directed to the corresponding author.

## Author contributions

CC: Conceptualization, Data curation, Formal Analysis, Investigation, Methodology, Resources, Software, Visualization, Writing – original draft, Writing – review and editing. MK: Conceptualization, Methodology, Project administration, Supervision, Visualization, Writing – review and editing, Validation, Formal Analysis, Investigation. AA: Conceptualization, Data curation, Funding acquisition, Methodology, Project administration, Resources, Supervision, Validation, Visualization, Writing – review and editing.
